# The Significance of a Mushroom Diet in the Prevention of Osteoporosis

**DOI:** 10.3390/ph19030482

**Published:** 2026-03-15

**Authors:** Małgorzata Cicha-Jeleń, Katarzyna Kała, Katarzyna Sułkowska-Ziaja, Bożena Muszyńska

**Affiliations:** 1 Department of Medicinal Plant and Mushroom Biotechnology, Faculty of Pharmacy, Jagiellonian University Medical College, 9 Medyczna, 30-688 Kraków, Poland; katarzyna.sulkowska-ziaja@uj.edu.pl (K.S.-Z.); bozena.muszynska@uj.edu.pl (B.M.); 2 Doctoral School of Medical and Health Sciences, Faculty of Pharmacy, Jagiellonian University Medical College, 16 św. Łazarza, 31-530 Kraków, Poland

**Keywords:** osteoporosis, bone regeneration, vitamin D_2_, calcium, medicinal mushrooms

## Abstract

Osteoporosis is a metabolic disease of the skeleton characterized by a low bone mass and deterioration of bone tissue structure, leading to increased fragility and susceptibility to fractures. It is often referred to as the “silent killer of bones” because it progresses without symptoms until a bone fracture occurs. Osteoporosis is a serious health problem, especially in aging societies, leading to fractures, limited mobility, and a decreased quality of life. Osteoporosis prevention through dietary modification should be the first step in protecting bone health before implementing any form of pharmacotherapy. The composition of the diet and nutritional patterns are considered the most important factors influencing the shaping of gut microbiota and its metabolites, which in turn affect the regulation of bone tissue metabolism. Mushrooms, as a source of vitamin D, can play a significant role in the prevention of osteoporosis. Additionally, the application of UV irradiation can rapidly increase the vitamin D_2_ content in mushrooms. A review of currently available studies reveals that many mushroom species contain substances (Ca, P, Se) that support bone formation by promoting remineralization. Mushrooms also induce bone regeneration after osteoporosis by balancing their reconstruction. This review systematically integrates the latest research on the use of mushrooms in the prevention of osteoporosis. The most promising species in the prevention of osteoporosis include: *Lentinula edodes*, *Ganoderma lucidum*, *Ophiocordyceps sinensis*, *Pleurotus eryngii*, *Antrodia camphorata*, *Auricularia auricula*, *Agaricus bisporus*, *and Grifola frondosa*.

## 1. Introduction

**Figure 1:**
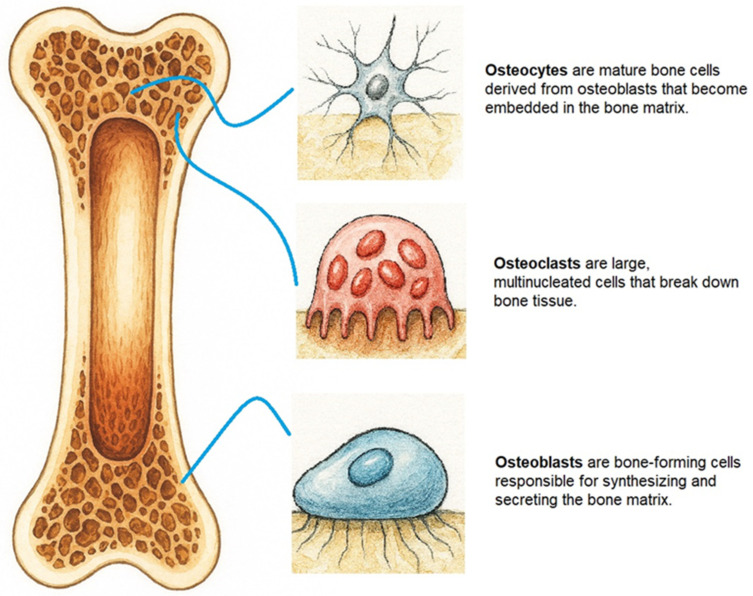
The main types of cells responsible for maintaining bone tissue.

Throughout our lives, our bones undergo continuous remodeling (degradation and regeneration), which is crucial for maintaining their shape and integrity. Bone remodeling is precisely regulated by osteoclasts and osteoblasts (Figure 1). Disruption of these processes leads to skeletal system disorders, such as osteoporosis [1,2]. Senescent bone marrow mesenchymal stem cell (BMSC) display diminished proliferative and differentiation capacities, which contribute to the development of osteoporosis and impair bone repair. Consequently, interventions aimed at mitigating BMSC senescence and restoring their regenerative potential are critically important [3].

According to the World Health Organization, osteoporosis is a condition marked by low bone mass and deterioration of the bone’s microarchitecture, which makes bones more fragile and increases the risk of fractures from minimal trauma. It is often called a “silent disease” because it progresses without symptoms until a fracture occurs [4].

Osteoporosis is a major health concern, especially in aging populations, because it raises the risk of fractures, limits mobility, and reduces quality of life. Recognized as a significant public-health issue, it affects over 200 million people worldwide. In the European Union, an estimated 22 million women and 5.5 million men have the condition. Existing drug treatments are linked to various side effects, highlighting the need to develop alternative therapeutic options [5,6,7,8,9].

The prevalence of osteoporosis shows regional heterogeneity, with the highest rates reported in postmenopausal women. This distribution is attributable to the postmenopausal decline in circulating estrogen levels, a hormone essential for the preservation of bone mass and microarchitectural integrity [9,10]. Osteoporosis is a metabolic bone disorder that frequently develops in patients subjected to prolonged bed rest. This increased susceptibility is attributable to the absence of weight-bearing mechanical loading on the limbs. For analogous reasons, exposure to microgravity leads to bone loss in astronauts, who experience reduced skeletal loading during spaceflight [11,12].

Based on bone metabolic mechanisms, clinical approaches to osteoporosis are categorized into three principal groups: antiresorptive agents that inhibit osteoclastic bone breakdown, anabolic therapies that stimulate new bone formation, and bone-mineralizing treatments that enhance mineral deposition and improve bone density [13].

Management of osteoporosis comprises both pharmacological and non-pharmacological interventions. Non-pharmacological strategies emphasize lifestyle modification and dietary optimization designed to enhance osteogenic processes and suppress bone resorption [5]. Before initiating pharmacotherapy, primary protection of skeletal health should prioritize dietary modification. Dietary composition and overall nutritional patterns are key determinants of gut microbiota structure and its metabolite profile, which in turn influence bone metabolism, including remodeling and mineralization [8,14].

Pharmacotherapy for osteoporosis principally comprises antiresorptive agents including bisphosphonates, estrogens, and selective estrogen receptor modulators (SERMs) such as raloxifene which remain the most frequently prescribed interventions to lower the incidence of primary and recurrent fractures in postmenopausal patients. The use of these agents is tempered by notable adverse effects, for example a potential elevation in breast cancer risk, which limits their acceptability in some patients [8,15,16]. The anabolic class is represented clinically by parathyroid hormone (PTH) analogs, exemplified by teriparatide, the sole anabolic agent currently approved by the US Food and Drug Administration. Its application is constrained by high cost and the requirement for daily subcutaneous administration, and treatment duration is typically restricted to 24 months, reserved for severe osteoporosis or glucocorticoid-induced cases [8,15,16].

Bisphosphonates mitigate bone loss by suppressing osteoclastic resorption. However, their utility is constrained because they do not promote new bone formation and are associated with adverse effects that range from mild gastrointestinal disturbances, musculoskeletal pain, and influenza-like symptoms to rare but serious complications such as osteonecrosis of the jaw. Cell-based therapies hold therapeutic promise but face significant challenges, including poor homing of mesenchymal stem cells to skeletal sites and uncertain post-implantation cell fate. Additional supportive measures include calcium and vitamin D supplementation to optimize mineral availability for bone maintenance [10].

Calcium and vitamin D have long been recognized as fundamental constituents of bone remodeling, and their supplementation is a standard adjunct in osteoporosis management. However, supplementation alone yields limited efficacy and is typically combined with other therapeutic modalities. Widely used calcium carbonate supplements exhibit suboptimal bioavailability. Emerging evidence indicates that potassium may contribute importantly to the maintenance of bone mineral density (BMD) and the prevention of osteoporosis [12,17,18].

The aim of our manuscript was to compile and synthesize evidence on the anti-osteoporotic mechanisms of selected edible mushrooms species.

## 2. Research Selection Criteria

**Figure 2:**
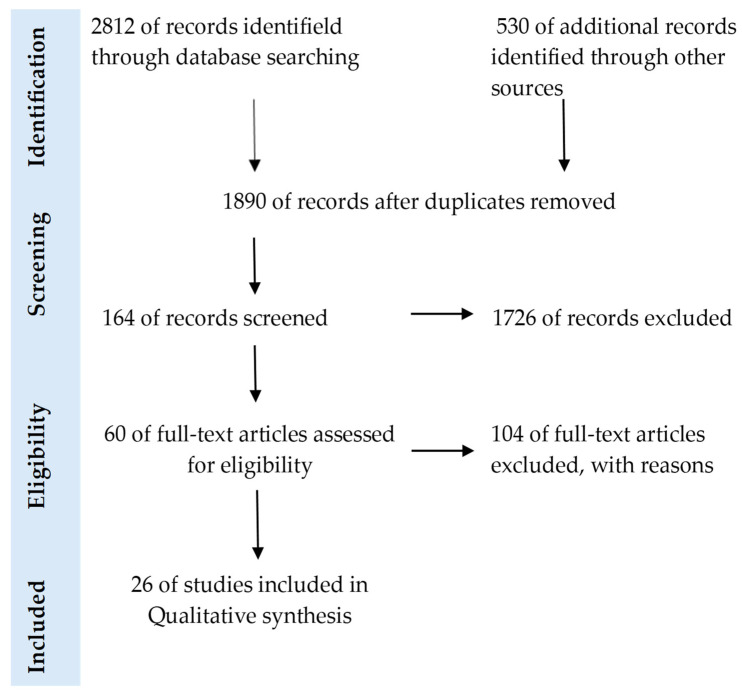
PRISMA flow diagram.

This review was conducted between September 2024 and January 2026 using the PubMed and Embase database. To evaluate the available literature, the following search terms were applied: “mushrooms”, “medicinal mushrooms”, “edible mushrooms”, “ergosterol”, “vitamin D_2_”, “osteoporosis”, bone mineral density”, “bone health”, “bone metabolism”, “vitamin D metabolism”, “gut microbiota”. The keywords were combined using Boolean operators (‘AND’ and ‘OR’). When searching, it also uses additional search sources from Google Scholar. The preliminary search yielded 3342 results (*n* = 3342). We identified 26 studies for full-text, requesting detailed descriptions of the in vitro and in vivo experiments conducted on individual edible mushroom species. We did not apply any criteria related to language or publication year. In the PubMed database, we additionally filtered the search according to two criteria. The first was “Text Availability”, including only “Free Full Text” or “Full Text” publications. The second was “Article Type”, including Books and Documents, Classical Article, Clinical Study, Clinical Trial, Clinical Trial, Veterinary, Controlled Clinical Trial, Meta-Analysis, Randomized Controlled Trial, Randomized Controlled Trial, Veterinary, Review, Systematic Review, and Article, review and clinical trial in Embase. Details on all records identified, abstracts and full-text articles assessed, along with the studies excluded and those incorporated into the qualitative and quantitative analyses, are presented in the PRISMA flow diagram (Figure 2). To enhance the precision of the search strategy, both MeSH (Medical Subject Headings) descriptors and non-MeSH keywords were employed, together with appropriate Boolean combinations [19,20].

## 3. Vitamin D in Mushrooms

Natural compounds that inhibit osteoclast differentiation are increasingly being adopted within current therapeutic strategies to enhance skeletal health in osteoporosis, owing to their potential to reduce bone resorption and complement existing treatments [1].

Medicinal mushrooms exhibit a broad spectrum of bioactive properties, encompassing anti-allergic, antibacterial, antifungal, antioxidant, anti-inflammatory, antiviral, and anticancer activities, as well as immunomodulatory effects. They have also been reported to exert antihyperlipidemic, antidiabetic, gastroprotective, hepatoprotective, antidepressant, neuroprotective, nephroprotective, and antihypertensive actions, and notably possess osteoprotective potential [21,22].

In the context of discussing the health-promoting properties of mushrooms, it is also essential to consider their capacity to accumulate harmful metals. Mushrooms are capable of accumulating very high levels of both harmful trace elements and radioactive cesium isotopes when growing on heavily contaminated substrates, in the vicinity of metal smelters, in mining areas, along high-traffic roads, or within urban environments. Most wild mushrooms collected from uncontaminated areas do not pose a health risk. In the case of cultivated mushrooms, the risk is minimal, as they are grown on controlled, clean substrates, which results in low concentrations of hazardous elements [23]. Studies have also been conducted to determine the levels of cadmium and lead ions in edible mushrooms collected from natural habitats. The extent of heavy-metal release from mushroom fruiting bodies into artificial digestive juices was assessed under conditions simulating the human gastrointestinal tract. Following extraction of mushroom material into artificial digestive fluids, the concentrations of cadmium and lead were found to be well below the permissible intake limits for humans. Additionally, experiments were performed to evaluate the release of metals into artificial digestive juices from in vitro cultures grown on media enriched with cadmium and lead ions. These studies demonstrated high accumulation of both metals in the fungal biomass, accompanied by only minimal release into artificial digestive fluids [23].

Vitamin D derived from mushrooms exerts well-established effects on skeletal physiology and additionally confers multiple systemic health benefits. Achieving adequate vitamin D status through diet alone is challenging, since naturally rich sources are largely confined to oily fish and fish liver oils. Consequently, several countries implement food fortification programs, adding vitamin D to staples such as fruit juices, milk, selected breakfast cereals, and yogurt to improve population intake [24]. In Finland, voluntary fortification of milk and spreadable fats with vitamin D was associated with an increase in mean population serum 25(OH)D concentrations. In the United States, fortified milk and dairy products contribute the largest proportion of dietary vitamin D intake (43.7%), and UV-exposed mushroom powder has been approved by the US Food and Drug Administration (FDA) as a source of vitamin D_2_ for incorporation into selected food products [25,26]. Dried mushroom powder represents a promising natural alternative for vitamin D fortification of foods, with potential to mitigate population-level vitamin D deficiency and thereby reduce osteoporosis risk. In one study, dried mushroom powder (DMP) prepared after 60 min of sunlight exposure was incorporated as a vitamin D source into selected food matrices (waffles, breadsticks, and salad dressing). Addition of the UV-exposed mushroom powder increased the products’ protein, fat, and vitamin D contents while reducing carbohydrate proportion, and also enhanced their antioxidant activity. The authors reported that consumption of 100 g portions of these products containing 3% DMP (sunlight-exposed for 60 min) supplied the full or nearly full recommended daily intake of vitamin D [26,27].

**Figure 3:**
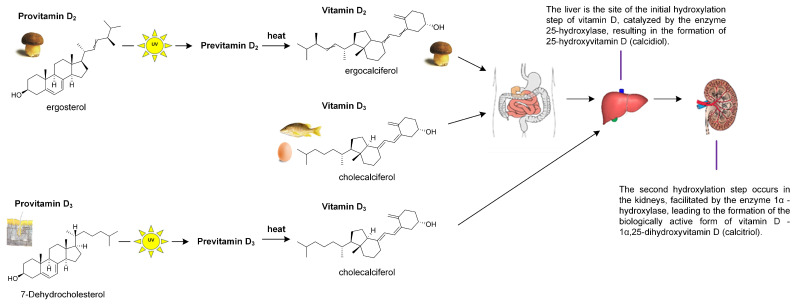
Schematic representation of vitamin D metabolic pathways in the human body.

Nevertheless, dietary measures alone are insufficient to avert vitamin D deficiency, prompting the exploration of alternative strategies to achieve adequate intake. Mushrooms represent one of the limited non-animal reservoirs of the vitamin D_2_ precursor ergosterol, which is metabolically converted in humans to biologically active vitamin D, as illustrated in Figure 3 [28].

Exposure of mushrooms to ultraviolet radiation, whether solar or artificial, provides a practical alternative source of vitamin D by photoconverting the sterol precursor ergosterol into ergocalciferol (vitamin D_2_). Preclinical studies in animal models demonstrate that consumption of UV-treated mushrooms elevates circulating total 25–hydroxyvitamin D [25(OH)D] concentrations, a biochemical change that is expected to translate into improved clinical outcomes associated with vitamin D activity [25,29,30,31,32]. Vitamin D_2_ derived from edible mushrooms exposed to UVB radiation is both bioavailable and safe. In an experimental study, ovariectomized mice fed UV-pulsed mushrooms demonstrated increased BMD and distinct alterations in serum metabolite profiles relative to ovariectomized controls receiving standard chow. These results are indicative of enhanced skeletal health and imply potential translational relevance for human osteoporosis prevention [33,34]. In the conducted analyses, administration of UVB-irradiated mushrooms for four weeks to vitamin D-deficient rats produced significant improvements in serum biomarkers, including 25–hydroxyvitamin D (25(OH)D), PTH, calcium, phosphorus, and alkaline phosphatase (ALP). Histomorphometric examination of the femur revealed enhanced osteoid formation and a decrease in trabecular separation, indicating amelioration of bone microarchitecture [15,35]. In animals receiving vitamin D-fortified mushrooms, hepatic expression of CYP2R1 and VDR and renal expression of CYP27B1 and VDR were upregulated. This transcriptional response reflects enhanced vitamin D biotransformation and signaling, which could translate into improved skeletal health and systemic well-being. CYP2R1 is an enzyme that converts vitamin D into its active form. Vitamin D Receptor (VDR) is the receptor for vitamin D, which binds to its active form, allowing it to act at the cellular level. CYP27B1 is an enzyme that converts 25(OH)D into its most active form, 1,25–dihydroxyvitamin D [15,35].

## 4. Selected Mushroom Species and Their Relevance in the Treatment of Osteoporosis

### 4.1. Selected Mushrooms Species

Below are described the species of mushrooms that have been used in studies to test their potential effect in preventing osteoporosis. All of the mushroom species presented are suitable for cultivation, which significantly facilitates the potential future acquisition of raw material.

#### 4.1.1. *Lentinula edodes*

*Lentinula edodes* (shiitake) is among the most widely cultivated edible fungi worldwide and demonstrates a broad spectrum of bioactivities, including antineoplastic, antioxidant, antiviral, immunomodulatory, and antimicrobial effects [1]. In a study, the ethyl acetate extract of *L. edodes* (LEA) markedly inhibited osteoclastogenesis by attenuating receptor activator of NF–κB ligand (RANKL)-stimulated induction of nuclear factor-activated T cells c1 (NFATc1), the master transcriptional regulator of osteoclast differentiation [1,36].

In an experimental study by Dong Jae Won et al., ovariectomized and sham-operated rats were fed either UVB-irradiated or non-irradiated *L. edodes* powder. Animals receiving the UVB-treated shiitake exhibited significantly higher circulating 25–hydroxyvitamin D (25(OH)D) concentrations, indicating efficient absorption of mushroom-derived vitamin D_2_ compared with controls [32]. Exposure of *L. edodes* fruiting bodies to ultraviolet radiation increases their vitamin D_2_ content and alters cellular architecture, thereby enhancing calcium bioavailability relative to non-irradiated mushrooms. Consequently, such UV-treated mushrooms have been proposed as a natural dietary source of vitamin D [37,38]. Another study investigated whether supplementation with modified *L. edodes* fruiting bodies could protect against bone deterioration in mice fed a diet low in calcium and lacking vitamin D_3_. Male mice aged 4–8 weeks were given diets supplemented with 5, 10, or 20% of either native, calcium-fortified, or calcium plus vitamin D_2_-fortified shiitake for four weeks. The investigators measured the femur density and length, expression levels of active calcium transport genes, and serum calcium. Animals on the deficient diet developed osteoporosis-like signs within four weeks, while those fed calcium and vitamin D_2_-enriched mushrooms showed significantly higher femoral density and thicker tibiae [38]. The expression of genes mediating active calcium transport in the duodenum and kidney was markedly upregulated. These findings suggest that vitamin D_2_ and/or calcium derived from UV-irradiated *L. edodes* enhance bone mineralization both by direct effects on bone tissue and by stimulating intestinal and renal calcium-absorptive pathways. Control animals fed standard chow exhibited significantly greater femoral length than mice maintained on a low-calcium, vitamin D_3_-deficient diet regardless of supplementation, indicating that while calcium- and vitamin D_2_-fortified mushrooms increase BMD, they do not influence longitudinal bone growth [38]. Moreover, BMD in mice receiving only calcium-enriched *L. edodes* was lower than in those given mushrooms enriched with both calcium and vitamin D_2_ or fed a standard diet, implying that concurrent enrichment with vitamin D_2_ is required to ameliorate osteoporosis-like changes in this model [38].

**Figure 4:**
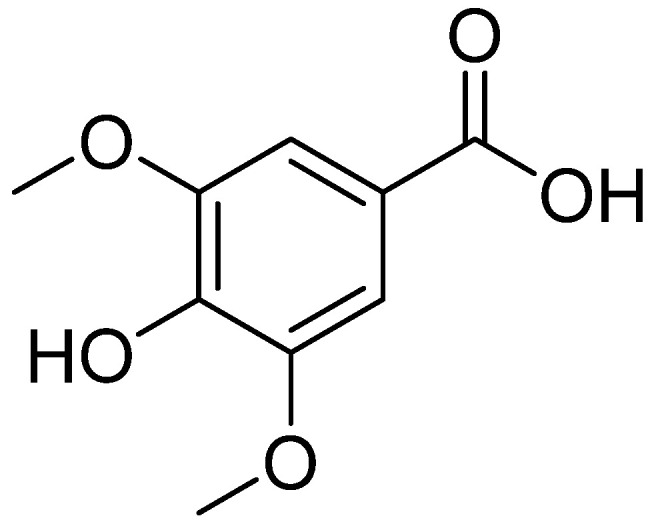
Chemical structure of syringic acid.

One study evaluated the chemopreventive potential of syringic acid (SA), a phenolic compound found, among other sources, in shiitake mycelium, in a model of postmenopausal osteoporosis (Figure 4). Experiments were conducted in OVX mice that received a diet supplemented with SA for a defined period. Bone parameters, biochemical markers of bone metabolism, and histological changes were subsequently assessed. Methods included comparison of three groups: OVX mice on a control diet, OVX mice on an SA-supplemented diet, and a non-OVX control group. The results indicate that the SA-supplemented diet significantly attenuated bone loss in OVX mice: treated animals exhibited higher bone mineral density and improved microarchitectural parameters compared with OVX controls. In addition, SA reduced markers of bone resorption, suggesting inhibition of osteoclast activity, while exerting favorable effects on indices of bone formation. Histological analyses corroborated improvements in bone structure in SA-treated animals. Importantly, SA showed no affinity for estrogen receptors, implying that its anti-osteoporotic effect is not mediated by estrogenic activity and may therefore present a lower risk profile relative to hormone-based therapies [2,39].

#### 4.1.2. *Grifola frondosa*

*Grifola frondosa* (maitake) grows on deciduous trees, especially oaks and beeches and displays multiple bioactivities such as anticancer, immune-supportive, and antidiabetic properties. In light of its beneficial effects on diabetes and the recognized connection between diabetes and bone disease, one study posited that *G. frondosa* could have a direct impact on bone cell biology [40]. An in vivo investigation linked type 1 diabetes to impaired osteoblast function and identified bone mass loss as a late diabetic complication. Insulin was shown to stimulate osteogenesis by enhancing both matrix synthesis and mineral deposition. Given the epidemiological and mechanistic association between diabetes and osteoporosis, it was hypothesized that an antidiabetic nutraceutical might confer skeletal protection [41]. To test this, researchers evaluated an aqueous extract of *G. frondosa* for osteoinductive activity on two human osteoblastic osteosarcoma lines (HOS58 and SaOS–2), using alkaline phosphatase (ALP) activity and matrix mineralization as markers of osteoblastic viability and maturation. Exposure of HOS58 cells to *G. frondosa* extract (0.8–120 µg/mL) for 5 days produced a significant elevation in ALP activity versus untreated controls, and SaOS–2 cells treated with *G. frondosa* extract (20 µg/mL) for 21 days exhibited nearly a twofold increase in mineralization compared with β-glycerophosphate-treated controls [41]. Collectively, the data indicate that *G. frondosa* aqueous extract acts as an osteogenic stimulus in human osteoblast cultures, elevating ALP activity and promoting mineral deposition when applied at concentrations below 30 µg/mL. The pronounced biological activity at these modest doses suggests active compounds that enhance bone formation and mineralization, consistent with a prospective role for the extract in preventing osteoporosis and attenuating diabetes-related bone pathology [41]. In one study, combined extracts of *L. edodes* and *G. frondosa* attenuated trabecular bone loss in the lumbar spine. The anti-osteoclastic activity of *L. edodes* extracts and their in vitro promotion of osteoblastic function, together with the observed reduction in spinal bone loss in an osteoporosis animal model, support the consideration of medicinal mushroom extracts as preventive agents or adjuncts to conventional pharmacotherapy to improve efficacy and reduce adverse effects. Bone loss was quantified by dual-energy X-ray absorptiometry (densitometry) and micro-computed tomography, and both *G. frondosa* and *L. edodes* extracts significantly decreased the number and activity of mature osteoclasts. Nevertheless, densitometric analysis revealed a 2.5% decline in BMD in ovariectomized rats, and no significant preservation of femoral bone volume or lumbar spine BMD was detected with mushroom extract treatment [42]. An aqueous extract of *L. edodes* markedly enhanced osteoblastic differentiation in vitro, producing a significant elevation in alkaline phosphatase activity and approximately a twofold increase in matrix mineralization. Similarly, low-dose aqueous extracts of *G. frondosa* and *L. edodes* augmented ALP activity and promoted bone mineral deposition. The bioactive constituents responsible for these osteogenic effects have not been isolated but are likely fungal polysaccharides, especially β-glucans [42].

#### 4.1.3. *Grifola gargal*

**Figure 5:**
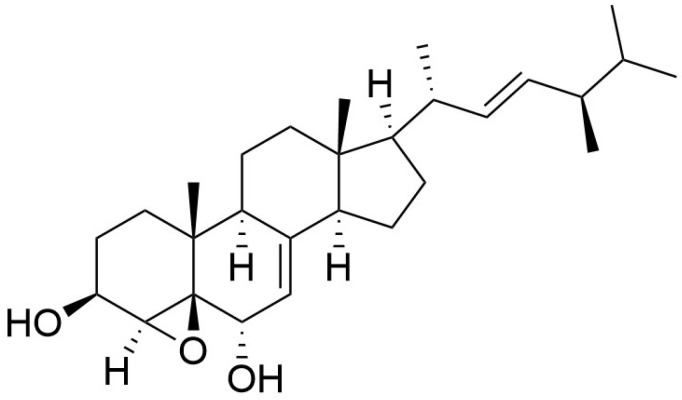
Chemical structure of gargalol A.

**Figure 6:**
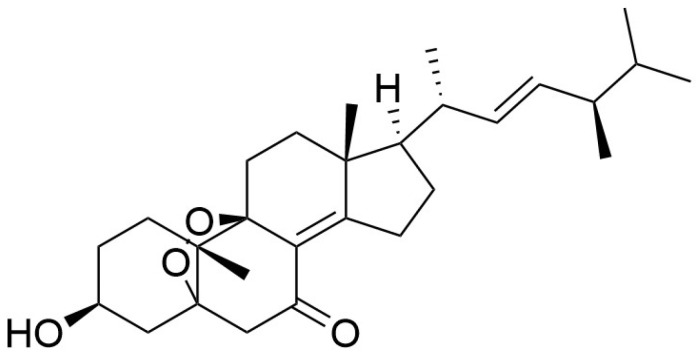
Chemical structure of gargalol B.

**Figure 7:**
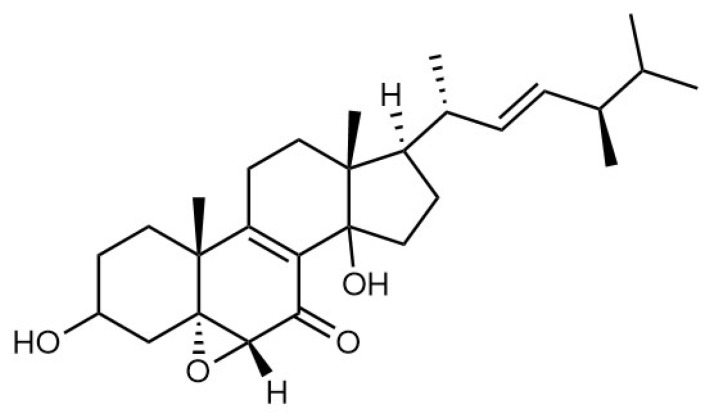
Chemical structure of gargalol C.

*Grifola gargal* has emerged as a noteworthy source of bioactive compounds with potential medicinal and functional applications. This edible mushrooms, characterized by its distinctive almond-like flavor, is traditionally collected and consumed by indigenous communities in southern Argentina and Chile. Aqueous extracts derived from this species demonstrate significant antioxidant and anti-inflammatory activities [43]. One investigation reported that *G. gargal* exhibits bioactivity relevant to anti-aging and osteoporosis prevention. The study assessed serum markers reflective of osteoblastic activity and osteoclastic resorption. Changes in these markers indicate that *G. gargal* may influence the balance between bone formation and bone resorption. Short-term administration of *G. gargal* powder (GGP) was associated with a significant reduction in deoxypyridinoline (DPD), a biomarker of bone resorption. Consequently, GGP and its constituent compounds, gargalols A–C (Figure 5, Figure 6 and Figure 7) may effectively attenuate bone resorption to mitigate osteoporosis risk. These compounds have been previously reported to suppress osteoclast formation in vitro, indicating that the mushroom may exert antiresorptive effects by interfering with osteoclast differentiation pathways. Moreover, *G. gargal* contains substantial amounts of vitamin D (236–258 μg/100 g GGP) and ergosterol (271–939 μg/100 g GGP) [43]. Given this background, the reduction in bone resorption markers observed in the human study may reflect inhibition of osteoclastogenesis or decreased osteoclast activity. Conversely, improvements in formation markers may indicate enhanced osteoblast function or increased matrix synthesis, potentially mediated by polysaccharide-driven immunomodulatory or anabolic effects [43].

Taken together, the findings suggest that *G. gargal* may influence bone metabolism through a combination of modulation of bone turnover markers, indicating shifts in osteoblast and osteoclast activity, presence of osteoclast suppressing compounds such as gargalols, which may reduce bone resorption and improvement of serum lipid profiles, which may indirectly support bone integrity by reducing metabolic and inflammatory stress.

Although the study does not delineate molecular pathways, the pattern of biomarker changes aligns with a model in which *G. gargal* contributes to a more balanced bone remodeling environment, favoring skeletal maintenance in aging individuals [43].

#### 4.1.4. *Ganoderma lucidum*

*Ganoderma lucidum* (reishi) is a medicinal mushroom long employed in traditional Chinese medicine, typically consumed as infusions and as powdered dietary supplements. Hyperactivation of osteoclasts driven by elevated receptor activator of nuclear factor–κB/receptor activator of nuclear factor–κB ligand (RANK/RANKL) signaling promotes osteolytic bone disorders, including osteoporosis and rheumatoid arthritis. Accordingly, pharmacological modulation of the RANK/RANKL axis using natural products, particularly medicinal mushrooms, constitutes a promising strategy for the treatment of pathological bone loss [44,45]. Ethanolic extracts of *G. lucidum* mitigate ovariectomy-induced bone loss arising from estrogen deficiency. They may also lower circulating osteocalcin concentrations, an effect comparable to that elicited by 17β–estradiol [4,45]. The anti-osteoporotic activity of *G. lucidum* is closely linked to the inhibition of osteoclastogenesis within osteoclasts [13].

**Figure 8:**
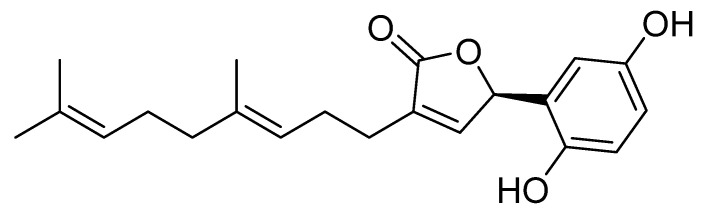
Chemical structure of ganomycin I.

**Figure 9:**
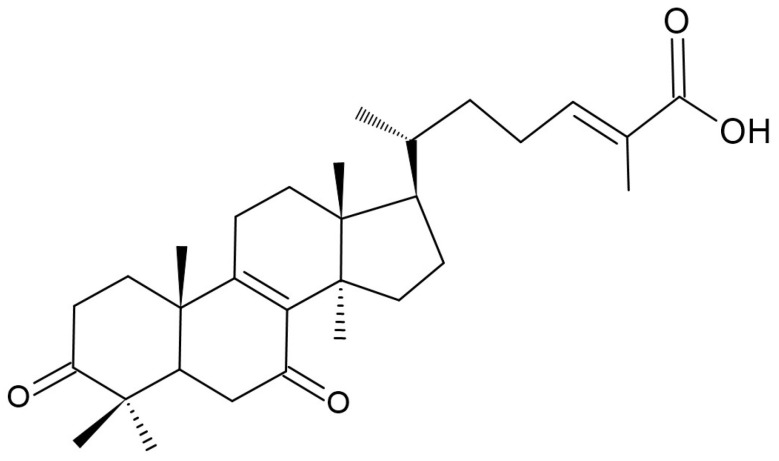
Chemical structure of ganoderic acid DM.

Ganomycin I (GMI) (Figure 8), a meroterpenoid derived from *G. lucidum*, suppresses RANKL-driven osteoclastogenesis. GMI markedly attenuates RANKL-induced osteoclast differentiation by decreasing osteoclast numbers, actin ring formation, and bone resorptive activity in a dose-dependent manner, without compromising cell viability, underscoring its potential benefit for human health [45,46]. GMI disrupts RANKL triggered signaling by inhibiting phosphorylation of the mitogen-activated protein kinase (p38 MAPK), extracellular signal-regulated kinase (ERK), c–Jun N–terminal kinase (JNK), and by suppressing induction of the osteoclastogenic transcription factors c–Fos and NFATc1, thereby impeding osteoclast differentiation. In RANKL activated bone marrow-derived macrophages (BMM), GMI markedly diminishes transcriptional and/or protein levels of osteoclast, specific markers such as proto-oncogene tyrosine-protein kinase Src (c–Src), tartarate-resistant acid phosphatase (TRAP), cathepsin K (CtsK), osteoclast-associated receptor (OSCAR), matrix metallopeptidase 9 (MMP–9), and dendritic cell-specific transmembrane protein (DC–STAMP), thereby impairing the osteoclastogenic program [45,46]. Triterpenoids constitute a major class of secondary metabolites in *G. lucidum*, with more than 120 distinct chemical structures having been characterized to date [13]. Ganoderic acid DM (Figure 9), a triterpenoid from *G. lucidum*, inhibits osteoclastogenesis in bone marrow cells and RAW 264D (RAWD) cell clones, which is an Abelson leukemia virus transformed line by downregulating the osteoclastogenic transcription factors c–Fos and NFATc1, an effect comparable to that of ganomycin. Consequently, ganoderic acid DM suppresses DC–STAMP expression and diminishes osteoclast fusion [22,46,47]. Ethanolic extracts of *G. lucidum* were evaluated for their ability to prevent ovariectomy (OVX) induced declines in BMD using 11-week-old female Sprague-Dawley rats. OVX animals receiving *G. lucidum* treatment exhibited significantly improved bone density relative to untreated OVX controls. Furthermore, the ethanolic extracts inhibited osteoclast differentiation stimulated by receptor activator of RANKL and tumor necrosis factor–α (TNF–α) [47]. Ethanolic extracts of *G. lucidum* fruiting bodies markedly promoted proliferation of MCF–7 breast cancer cells. This proliferative effect is attributed to the estrogenic activity of the extract and was abolished by the anti-estrogen ICI 182,780 [3].

*G. lucidum* promotes bone formation and inhibits bone resorption in part by modulating gut microbiota homeostasis and attenuating systemic inflammation. In a review by Chen et al., it was reported that the addition of *G. lucidum* to rice wine followed by storage in a porcelain vessel for one week prior to consumption, or boiling the fungus in water and incorporating the resulting decoction into rice porridge, produced osteoprotective effects. Clinical studies have confirmed that *G. lucidum*, as a medicinal mushroom, is non-toxic, does not elicit significant adverse effects, and exhibits therapeutic activity across a range of disorders [48].

In one study, OVX mice received diets supplemented with *L. edodes* fermented okara (LEFO) or *G. lucidum* fermented okara (GLFO). Okara, a soy processing byproduct, is frequently discarded in industry, generating economic losses and socio-environmental challenges. Consequently, strategies to repurpose and add value to such byproducts have attracted global interest. Framed within circular-economy principles, solid-state fermentation of okara with *G. lucidum* or *L. edodes* markedly improves its nutritional profile, and microbial biotransformation confers additional health-promoting and organoleptic enhancements to the raw substrate [49]. Using these fermented okara products prophylactically may offer a safe and effective alternative to standard osteoporosis treatments. LEFO and GLFO are rich in complex triterpenoids, polysaccharides (including β-D-glucans and chitosan derivatives), and ergosterol, all of which promote osteogenic differentiation. Triterpenoids in particular exert a protective influence on bone remodeling and show promise for both prevention and therapy of osteoporosis [49]. GLFO suppressed the OVX induced increase in osteocalcin, consistent with inhibition of osteoclast-driven bone loss. Micro CT and plasma biomarker evaluations in the OVX model revealed that GLFO improved bone outcomes more effectively than both raw okara and LEFO, and fermentation by *G. lucidum* or *L. edodes* was shown to modify okara composition and potentiate its anti-osteoporotic activity [49].

#### 4.1.5. *Ganoderma sinense*

*Ganoderma sinense* (black reishi) is traditionally employed in China to prevent and treat a range of disorders by modulating immune responses, with efficacy demonstrated in both in vivo and in vitro models. The aqueous extract of *G. sinense* fruiting bodies accelerates fracture repair, promoting disappearance of the fracture line, reconstruction of the medullary cavity, and near complete union. These outcomes are associated with modulation of calcium and phosphorus balance and alkaline phosphatase activity, which together enhance cartilage bridging [13].

#### 4.1.6. *Ophiocordyceps sinensis*

*Ophiocordyceps sinensis* (Chinese caterpillar fungus) has long been employed in China as a tonic to promote longevity, enhance endurance, and increase vitality. It is also incorporated into cosmetic formulations for its purported toning effects, and multiple studies report that *O. sinensis* improves insulin sensitivity and reduces plasma cholesterol concentrations [50]. On the other hand, the relationship between diabetes and decreased BMD has been confirmed in adults. BMD appears to be reduced both in the spine and hip in patients with diabetes. Consequently, there is an increased risk of fractures in individuals with diabetes and an increased risk of diabetes in individuals with osteopenia. Bone mineral content (BMC) in studied groups of diabetic patients is significantly decreased compared to the results obtained for control groups [50]. Administration of cordymin (a peptide isolated from *O. sinensis*) at 100 and 50 mg·kg^−1^·day^−1^ to alloxan-induced diabetic animals produced a significant increase in BMC relative to untreated diabetic controls. Moreover, the 100 mg·kg^−1^·day^−1^ dose significantly elevated femoral BMD in diabetic rats. The anti-osteopenic action of cordymin appears to be both direct via suppression of ALP and TRAP activities and indirect, through β-cell regeneration and consequent reductions in serum glucose and oxidative stress [50].

In another study, a polysaccharide-protein complex derived from *O. sinensis* was conjugated with selenium nanoparticles to yield a well-defined, highly stable SeNP formulation (Cs4–SeNP). While selenium nanoparticles display notable bioactivity and low toxicity, their impact on skeletal health has been scarcely investigated. Selenium, an essential trace element, is known to contribute to bone physiology [8,30]. However, the narrow therapeutic index of selenium complicates its clinical use. Over the past decades, selenium deficiency has been linked to deterioration of bone microarchitecture and to conditions such as osteopenia, osteoarthropathy and osteoporosis [8]. Studies have shown that Cs4–SeNP at 10 μM significantly promotes MC3T3–E1 cell proliferation, differentiation into osteoblasts, and subsequent mineral deposition. Once internalized by endocytosis, the complex stimulates oxidase 4 (Nox4)-derived reactive oxygen species (ROS) generation, which elevates bone morphogenic protein 2 (BMP–2) gene expression and triggers BMP signaling via canonical Smad pathways and a Smad-independent p38 MAPK route. In vivo, daily oral dosing of Cs4–SeNP (25–500 μg/kg body weight/day) for six weeks mitigates OVX-induced bone loss by stimulating bone formation, inhibiting osteoclastic activity, and restoring bone microstructure, indicating promise as a safe therapeutic nanomineral for postmenopausal osteoporosis [8,51].

Although all forms of osteoporosis arise from an imbalance between bone resorption and formation, the underlying causes differ: postmenopausal (primary type 1) osteoporosis is driven predominantly by hormonal changes, where estrogen deficiency (for example after menopause or ovariectomy) accelerates bone resorption and impairs new bone deposition, whereas osteoporosis due to prolonged immobilization or microgravity results from loss of mechanical loading that increases osteoclast activity, leading to reduced BMD and compromised bone microarchitecture [52]. In an in vivo study, it was reported that daily oral administration of *O. sinensis* to adult rats subjected to hindlimb suspension (HLS) prevented immobilization-induced bone loss and preserved trabecular microarchitecture. Animals were randomized into five groups: one HLS group received alendronate (2.0 mg·kg^−1^·day^−1^) as a positive control, three HLS groups received *O. sinensis* at 100, 300, or 500 mg·kg^−1^·day^−1^ administered orally for eight weeks before and after HLS, and one HLS group served as an untreated control. Alendronate sodium, a bisphosphonate and potent inhibitor of bone resorption, was included as a reference agent approved for several bone disorders including glucocorticoid-induced osteoporosis, male osteoporosis, and Paget’s disease [53]. Large clinical trials encompassing more than 17,000 subjects have demonstrated that alendronate increases bone mass and lowers the incidence of osteoporotic fractures. However, despite the potent antiresorptive action of bisphosphonates, they do not exert a direct anabolic effect on bone formation [53]. In this animal study, groups of ten males and ten females were evaluated for body weight, serum and urine biochemistry, BMD, BMC, trabecular microarchitecture, and biomechanical properties. Administration of *O. sinensis* at 300 and 500 mg·kg^−1^·day^−1^ or alendronate produced favorable effects on body mass, mechanical strength, BMD and BMC relative to untreated HLS groups. *O. sinensis* produced dose-dependent reductions in bone turnover markers and increased circulating osteocalcin in HLS rats. Micro-CT of the L4 vertebra demonstrated that 500 mg·kg^−1^·day^−1^ *O. sinensis* prevented loss of bone volume, enhanced connectivity density, and increased trabecular thickness and number, indicating that eight weeks of high-dose treatment can avert immobilization-induced osteoporosis and may have translational potential for humans. The bone-protective effects are likely mediated in part by antioxidant constituents of *O. sinensis* (cordycepic acid, vitamins C and E, polysaccharides, sterols), since elevated oxidative stress exacerbates resorption [53].

In separate experiments, rats received oral administrations of *O. sinensis*, strontium, or *O. sinensis* fortified with strontium ranelate (CSS), after which serum ALP, TRAP, osteocalcin (OC), homocysteine, C-terminal cross-linked telopeptides of type I collagen (CTX), estradiol, and interferon gamma (IFN–γ) were measured, CSS produced favorable effects on osteoporosis-related parameters, primarily through reductions in ALP, TRAP, CTX, and IFN–γ levels [54,55]. Concomitantly, CSS elevated serum OC and estradiol in ovariectomized rats with osteopenia. Current evidence indicates that strontium exerts metabolic actions on bone in vivo. Low-dose strontium administration has been shown experimentally to suppress bone resorption while stimulating bone formation, culminating in increased bone mass in animal models. Strontium ranelate, a semi-synthetic strontium salt, is the first anti-osteoporotic agent described to possess a dual mode of action simultaneously promoting bone formation and inhibiting resorption and its efficacy in reducing vertebral and hip fracture risk in postmenopausal osteoporosis has been demonstrated in multiple randomized controlled trials [54,55]. In animal models of confirmed osteoporosis CSS preserved bone mechanical properties and mineralization and counteracted bone loss, indicating that strontium-enriched *O. sinensis* may be a promising candidate for managing postmenopausal osteoporosis in women [54,55].

#### 4.1.7. *Cordyceps militaris*

**Figure 10:**
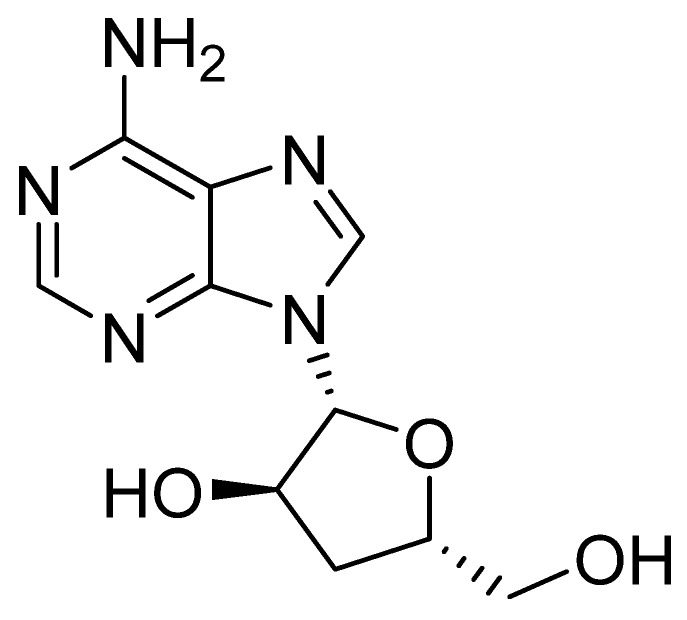
Chemical structure of cordycepin.

*Cordyceps militaris* exhibits a wide range of pharmacological activities, including anti-inflammatory, antioxidant, immunomodulatory, and anticancer properties, primarily associated with its bioactive compound cordycepin (a natural derivative of adenosine). In vitro assays revealed that cordycepin (Figure 10), isolated from *C. militaris* and applied at a concentration of 50 µg/mL, significantly suppressed ALP and TRAP enzymatic activity. Furthermore, in vivo studies conducted on rodent models demonstrated that oral administration of cordycepin effectively mitigated bone tissue degradation and enhanced the mechanical resilience of the femoral neck within an experimental osteoporosis model [6]. In RAW 264.7 macrophages, cordycepin produced a dose-dependent suppression of RANKL-driven osteoclast differentiation, concomitant with reduced mRNA expression of osteoclastogenesis-associated genes. The effects of the ethanol extract of *C. militaris* (CME) on bone loss were further assessed in vivo using a murine model of lipopolysaccharide (LPS)-induced bone resorption. Mice receiving CME exhibited markedly attenuated bone loss, indicating that cordycepin inhibits osteoclast differentiation in vitro and limits inflammatory bone degradation in vivo [56,57]. Wang et al. investigated cordycepin isolated from *C. militaris*, demonstrating that it mitigates bone loss and can delay cellular senescence induced by diverse pathological stimuli. In their study, combining dental pulp stem cell (DPSC)-derived exosomes with cordycepin enhanced the proliferation and osteogenic differentiation of senescent bone marrow-derived mesenchymal stem cells (BMSCs) and counteracted cellular senescence via activation of NRF2 signaling and preservation of heterochromatin stability. Injectable hydrogels enabling sustained release of the exosome-cordycepin complex effectively attenuated senescence and substantially accelerated regeneration and repair of aged bone tissue [3]. Collectively, these findings suggest that cordycepin may be beneficial in preventing osteoclast formation and the ensuing bone resorption that contributes to skeletal destruction [3,56,57].

#### 4.1.8. *Pleurotus eryngii*

*Pleurotus eryngii* (king oyster mushroom) exhibits hepatoprotective and nephroprotective effects and supports gastrointestinal health. Evidence from murine models indicates potential anticancer activity. In vitro, *P. eryngii* extracts suppress bone-resorbing osteoclasts while promoting the differentiation and activity of bone-forming osteoblasts. In vivo studies in OVX rat models of osteoporosis further demonstrate its anti-osteoporotic efficacy [58,59]. Oral administration of an aqueous *P. eryngii* extract at 0.4 mL/day for 4 weeks in bilaterally OVX rats enhanced osteoblast activity, evidenced by increased ALP activity and upregulated osteoprotegerin (OPG) gene expression, while concurrently suppressing osteoclast formation and function by reducing TRAP(+) multinucleated cells and resorption areas. Although the bioactive constituents of *P. eryngii* remain incompletely characterized, the findings suggest the presence of compounds capable of augmenting bone metabolism. In vivo, aqueous *P. eryngii* extracts (PEX) attenuated the loss of trabecular BMD in ovariectomy-induced osteoporotic rat models [60]. In a human osteosarcoma cell line (HOS cells) derived from adult bone tissue, PEX enhanced ALP activity, with significant elevations observed at 0.08 and 10 µL/mL. PEX also increased osteoprotegerin (OPG) secretion, with a 70% upregulation in OPG expression at 10 µL/mL. OPG suppresses osteoclastogenesis. Consistent with these effects, PEX markedly reduced the number of TRAP(+) multinucleated cells and resorption areas. In OVX rats on a standard diet, trabecular tibial BMD declined by approximately 16% over 4 weeks, whereas PEX-treated rats exhibited only an 8% reduction, indicating that PEX mitigates bone loss progression in OVX-induced osteoporosis [60].

#### 4.1.9. *Pleurotus ferulae*

*Pleurotus ferulae* is rich in phenolic and flavonoid compounds alongside other bioactive constituents that confer bone-protective effects. Osteoclasts, the cells responsible for bone resorption, serve as more sensitive early indicators of therapeutic response to osteoporosis interventions in postmenopausal women than markers of bone formation. Serum cross-linked N-telopeptide of type I collagen (NTX–I) and pyridinoline (PYD) are degradation products of type I collagen and thus reflect collagen breakdown and bone resorption. Mice treated with pulsed UV-irradiated *P. ferulae* (UV–PM) exhibited lower serum PYD and NTX–I concentrations compared with ovariectomized controls, suggesting that UV–PM may attenuate type I collagen degradation [33]. Mice fed UV–PM displayed significantly lower PYD levels compared with animals receiving non-irradiated *P. ferulae* (NPM), implying that the vitamin D_2_ generated by irradiation more effectively suppresses osteoclast activity than the unirradiated mushrooms. Animals given irradiated fruiting bodies containing vitamin D_2_ experienced reduced bone loss relative to those treated with non-irradiated material. NMR-based serum metabolomics revealed elevated concentrations of several amino acids in mice consuming irradiated *P. ferulae* fruiting bodies, including bone-relevant metabolites such as taurine, arginine, and lysine, which may contribute to the observed skeletal protective effects [33,61]. Taurine supplementation has been reported to increase BMD in rodent models. Mice administered irradiated *P. ferulae* exhibited higher serum taurine levels than those treated with alendronate, indicating that irradiation of *P. ferulae* may elevate concentrations of metabolites linked to skeletal health. Arginine contributes to the biosynthesis of substrates such as polyamines and proline, which are integral to collagen production, whereas lysine facilitates calcium absorption and promotes collagen cross-linking in bone. Additionally, metabolites associated with energy metabolism, including creatine and lactate, were increased in animals consuming irradiated *P. ferulae* fruiting bodies. Creatine supports bone formation and maintenance, has been shown to ameliorate osteoporosis in postmenopausal women, and can stimulate synthesis and secretion of type I collagen. It also provides an energy source that enables bone cells to survive, proliferate, differentiate, and produce extracellular matrix. Lactate may attenuate bone loss by participating in the regulation of collagen biosynthesis during osteogenesis [33].

#### 4.1.10. *Pleurotus ostreatus*

*Pleurotus ostreatus* (oyster mushroom) is recognized for a broad spectrum of health-promoting activities, including antioxidant, immunomodulatory, antihypertensive, hypocholesterolemic, antidiabetic, anti-inflammatory, and prebiotic effects. Modulation of the gut microbiota through mushroom-derived prebiotic compounds may represent a promising strategy to influence bone remodeling and thereby help prevent or mitigate bone-degenerative disorders [62]. One study investigated whether UVB irradiation could augment vitamin D_2_ content in *P. ostreatus* and evaluated the effects of the enriched mushrooms on dexamethasone-induced osteoporosis in mice. UVB treatment elevated the vitamin D concentration in the mushroom material. Mice fed with the vitamin D-supplemented mushroom powder exhibited increased numbers of osteocytes and osteoblasts and higher serum calcium levels, together with a reduction in osteoclast counts. These results indicate that vitamin D-fortified oyster mushrooms may exert protective, anti-osteoporotic effects [62]. Studies have demonstrated that protein nano-extracts derived from *P. ostreatus* have been shown to exert anti-inflammatory and anti-proliferative effects on macrophages, while concurrently providing cytoprotective benefits to healthy human osteoblasts. These findings indicate that such nano-formulations may modulate inflammatory responses and support osteoblast viability and function [63].

#### 4.1.11. *Pleurotus citripileatus*

*Pleurotus citrinopileatus* (golden oyster mushroom) is an edible mushroom with multiple health-promoting attributes. It exhibits strong antioxidant activity and contains bioactive constituents that may enhance immune function [64]. Extracts of *P. citrinopileatus* have demonstrated antiproliferative effects against cancer cell lines. The mushroom’s β-glucan fraction has been reported to suppress RANKL-stimulated osteoclast differentiation, identifying it as a promising source of bioactive agents for osteoporosis prevention and therapy. In particular, the water-soluble β-glucan preparation inhibits osteoclastogenic activity most effectively in the fraction with an average mass greater than 50 kDa [59,64,65]. Polysaccharide fractions obtained from *P. citrinopileatus* by ultrasonic extraction have been shown in vitro to modulate osteoblast proliferation in a concentration-dependent manner, concomitantly enhancing ALP activity [52].

#### 4.1.12. *Pleurotus sajor-caju*

*Pleurotus sajor-caju* (medicinal mushroom) exhibits neurotrophic, anticancer, antiviral, antibacterial, and cholesterol-lowering activities. A glucan oligosaccharide isolated from *P. sajor-caju* (Ps–GOS) has been reported to enhance osteoblast differentiation and mineralization. β-1,3-Glucanoligosaccharide fraction (Ps–GOS) exert multifaceted regulatory effects on bone remodeling, acting simultaneously on osteoblastogenesis and osteoclastogenesis through several converging intracellular signaling pathways. Ps–GOS, beyond its known neurotrophic, anticancer, antiviral, antibacterial, and cholesterol-lowering properties, demonstrates potent osteoanabolic and antiresorptive activities that are mechanistically well-supported by cellular studies [65,66]. In osteoblastic MC3T3–E1 cells, Ps–GOS enhances proliferation, promotes differentiation, and significantly increases matrix mineralization through coordinated activation of the BMP–2/Runx2/MAPK/Wnt/β-catenin signaling network. Upregulation of BMP–2 initiates osteogenic commitment, while increased expression and activity of Runx2—an essential transcription factor for osteoblast maturation—drives the expression of downstream osteogenic genes such as alkaline phosphatase, osteocalcin, and collagen type I. Concurrent activation of MAPK pathways (ERK, JNK, and p38) further amplifies osteoblast differentiation by stabilizing Runx2 and enhancing its transcriptional activity. In parallel, Ps–GOS stimulates the Wnt/β-catenin pathway, leading to β-catenin accumulation and nuclear translocation, where it promotes transcription of genes required for matrix formation and mineral deposition. These synergistic effects collectively shift osteoblasts toward a highly anabolic phenotype, resulting in robust mineralization [65,66]. Complementing its osteogenic actions, Ps–GOS also suppresses osteoclast differentiation and function. In RANKL-stimulated RAW 264.7 pre-osteoclastic cells, Ps–GOS inhibits the RANK/NF–κB/c–Fos/NFATc1 axis, which is indispensable for osteoclastogenesis. By attenuating NF–κB activation, Ps–GOS reduces the induction of c–Fos, thereby preventing the upregulation of NFATc1, the master transcription factor governing osteoclast formation, fusion, and resorptive activity. This inhibition leads to decreased expression of osteoclast-specific genes, including TRAP, cathepsin K, and MMP–9, ultimately impairing the ability of osteoclasts to degrade the bone matrix. The dual modulation of osteoblast and osteoclast pathways indicates that Ps–GOS not only promotes bone formation but also effectively restrains bone resorption [65,66]. Taken together, the combined activation of BMP–2/Runx2/MAPK/Wnt/β-catenin signaling in osteoblasts and the suppression of RANK/NF–κB/c–Fos/NFATc1 signaling in osteoclast precursors positions Ps–GOS as a potent regulator of bone homeostasis. Its capacity to simultaneously enhance osteogenesis and inhibit osteoclastogenesis highlights its therapeutic potential for conditions characterized by imbalanced bone remodeling, such as osteopenia and osteoporosis [65,66].

#### 4.1.13. *Antrodia camphorata*

*Antrodia camphorata* (camphor mushroom) possesses a range of well-documented medicinal activities, including a capacity to promote osteoblast differentiation. Traditional formulations derived from this species are employed for the management of gastrointestinal disorders, hypertension, and pain, and the mushrooms also demonstrates anti-inflammatory, antioxidant, and anticancer properties [17]. Ethanol extracts of *A. camphorata* (ACAE) promote bone regeneration by restoring balance between formation and resorption, supporting their potential as a safe and effective therapy for osteoporosis. *A. camphorata* is rich in bioactive components—polysaccharides, triterpenoids, ergosterol, calcium, germanium, phosphatase, and chitosan—that are implicated in driving osteogenic differentiation. Triterpenoids exert notable protective effects on bone remodeling in osteoporosis models, polysaccharides enhance osteogenesis, and ergosterol, as a precursor of vitamin D, stimulates osteoblastic differentiation [17]. ACAE enhances osteoblast differentiation and inhibits osteoclastogenesis in ovariectomized SAMP8 mice with accelerated aging by downregulating RANKL and markedly upregulating OPG. Treated animals showed improved BMD and reduced signs of osteoporosis, indicating a shift toward balanced bone remodeling. These findings align with prior evidence that triterpenoids can suppress osteoclast formation via RANKL reduction, suggesting that the triterpenoid fraction of ACAE may be a key mediator of its antiresorptive effects. Overall, the study demonstrated notable bone regeneration and attenuated bone loss, supporting ACAE as a promising candidate for osteoporosis prevention and potential therapy [17].

#### 4.1.14. *Auricularia auricula*

*Auricularia auricula* is highly prized across East Asia and has a long history in Traditional Chinese Medicine, where it has been used for millennia to treat postpartum weakness, muscle cramps, numbness, and pain resulting from injuries [12]. *A. auricula* enhances human immune function and displays anticancer, antiviral, antibacterial, and antiparasitic activities, reflecting a broad spectrum of bioactive effects [67]. Scientific evidence indicates that *A. auricula* lowers blood glucose and can alleviate symptoms of non-insulin-dependent diabetes, and it also has cholesterol-reducing effects. A peptide-calcium complex derived from *A. auricula* (AP–Ca) increased intracellular calcium content and alkaline phosphatase activity in osteoblasts in vitro, consistent with enhanced osteoblastic function [12,68]. In animal studies, AP–Ca improved bone quality by increasing BMD, mineral content, and organic matrix content. AP–Ca also downregulated pro-inflammatory genes associated with bone resorption. Overall, current research supports calcium supplementation as an effective strategy to reduce osteoporosis risk and maintain bone health, while noting that excessive calcium intake offers no added benefit and may be harmful [12,68]. The market offers inorganic and organic calcium supplements as well as amino acid-calcium complexes, but their intestinal absorption and bioavailability differ markedly. Inorganic and many organic forms often show limited therapeutic benefit because of poor uptake, whereas amino acid-calcium complexes are more stable and better absorbed, albeit typically more expensive. Peptide-calcium complexes represent a promising alternative, combining improved bioavailability with effective calcium delivery [12]. AP–Ca is absorbed by osteoblasts more readily than inorganic calcium, producing a stronger effect on enzymes involved in bone formation. AP–Ca protects against bone loss caused by prolonged immobilization by improving calcium delivery to sites of bone turnover and enhancing calcium absorption and utilization. In immobilization models, AP–Ca raised ALP activity and lowered serum TRAP levels, thereby stimulating bone formation while suppressing bone resorption. AP–Ca also reduced pro-inflammatory cytokine expression in immobilization-induced osteoporosis, helping to prevent further bone deterioration [12].

In the manuscript by Kim et al., the authors report investigations into the biphasic effects of an extract from *A. auricula* on bone homeostasis. Their objective was to determine whether the preparation could concurrently suppress excessive bone resorption and promote bone formation, thereby constituting a potential therapeutic candidate for prevention of bone loss. The study relied predominantly on in vitro models. The aqueous extract of *A. auricula* (AAJWE) inhibited the formation of multinucleated osteoclasts without exerting cytotoxic effects. The data indicate that AAJWE suppresses RANKL-induced osteoclastogenic markers, including c-Fos, TRAP, NFATc1, and cathepsin K. Moreover, AAJWE attenuated phosphorylation of ERK and JNK, upstream signaling molecules that modulate c-Fos and NFATc1 expression. These results suggest that AAJWE inhibits osteoclastogenesis by disrupting ERK and JNK signaling pathways and downregulating c-Fos and NFATc1 [69].

#### 4.1.15. *Agaricus bisporus*

**Figure 11:**
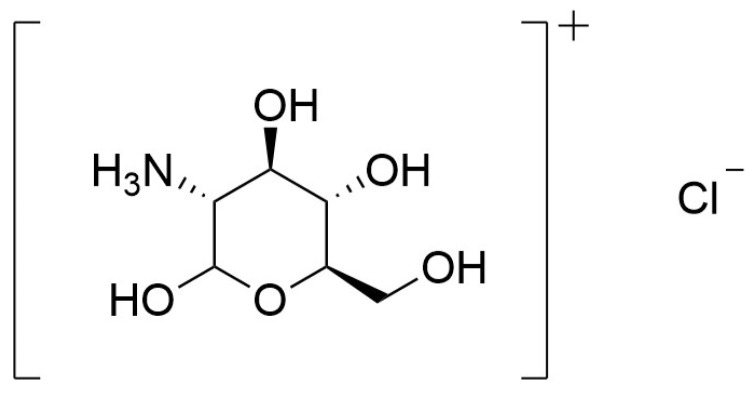
Chemical structure of glucosamine hydrochloride.

*Agaricus bisporus* (white button mushroom) is a source of ergothioneine, an antioxidant that helps shield cells from oxidative damage. The species also displays anti-inflammatory, immunostimulatory, and antimicrobial activities, contributing to its health-promoting profile [70]. In an experiment, powdered *A. bisporus* was added to the basal diet of *Coturnix coturnix japonica* at 0.25, 0.50, 0.75, or 1.00% and fed ad libitum for 42 days. Diets containing 0.50–0.75% mushroom powder (MP) improved certain outcomes, notably the oxidative stability of meat and some biomechanical bone properties. MP appeared most effective at enhancing growth and body weight during the early weeks, likely reflecting the developmental state of the quail intestine. Overall, MP was a safe feed ingredient that did not harm carcass traits or meat quality and produced measurable differences in performance between groups [70]. After three weeks, quails receiving 0.75% MP weighed more than those in the control group or the 0.25% group, while no difference was seen between control and 0.25% groups. MP supplementation did not significantly change cortical bone thickness, bone diameter, or cortical cross-section, but the 0.75% inclusion significantly increased bone shear strength compared with the control, 0.25%, and 1.00% groups [70]. In another study, glucosamine hydrochloride (GAH) (Figure 11) isolated from the cell wall of *A. bisporus* demonstrated a dose-dependent promotion of osteogenesis in zebrafish (*Brachydanio rerio*) larvae and enhanced caudal fin regeneration in adult *B. rerio*, indicating its promise as a candidate for osteoporosis prevention and treatment. GAH, a natural amino-monosaccharide derivative, supplies aminoglucose required for proteoglycan synthesis-key constituents of cartilage matrix that help absorb shock by limiting collagen fiber extension. Treatment with GAH upregulates bone-related marker genes, underscoring its role in skeletal development, and zebrafish studies indicate that GAH facilitates bone formation and repair via bone morphogenetic protein signaling [71].

In a six-week trial involving 40 healthy adults (aged 20–59 years), the bioavailability of vitamin D_2_ following consumption of *Agaricus bisporus* was evaluated. Participants were allocated to four groups: a control group receiving non-irradiated mushrooms; two experimental groups consuming UVB-irradiated mushrooms with differing vitamin D_2_ concentrations; and a comparator group receiving purified ergocalciferol together with non-irradiated mushrooms. Baseline serum 25(OH)D_2_ concentrations were low and comparable across groups. Intake of UV-irradiated mushrooms produced an increase in 25(OH)D_2_ in the experimental arms by week 3, with the greatest 25(OH)D_2_ levels observed in the group receiving purified ergocalciferol; levels in the control group remained low. However, total serum 25(OH)D did not improve in any treatment group relative to control, because the rise in 25(OH)D_2_ was counterbalanced by a significant decline in 25(OH)D_3_ observed in the treated cohorts over the study period. The authors conclude that ergocalciferol from UV-irradiated mushrooms is bioavailable (it elevates 25(OH)D_2_) but, in this study, did not translate into an improved overall vitamin D status due to the concurrent reduction in 25(OH)D_3_. These findings indicate the need for further research on the stability of mushroom-derived vitamin D_2_ and for long-term comparative studies of vitamin D_2_ versus D_3_ [31].

Starck et al. cited a study evaluating the effect of consuming UVB-irradiated *A. bisporus* on vitamin D status in humans. The trial enrolled healthy adult volunteers with low baseline vitamin D levels. The objective was to determine whether regular intake of mushrooms enriched in vitamin D_2_ could raise circulating 25(OH)D concentrations to an extent comparable with supplementation. For the intervention, portions of fresh mushrooms were briefly exposed to UVB radiation, increasing their vitamin D_2_ content to levels equivalent to a prophylactic supplement dose. Participants consumed one portion daily for several weeks; outcomes were compared with a control group receiving non-irradiated mushrooms and with a group receiving a purified ergocalciferol supplement [25]. At the end of the intervention, subjects who consumed UV-irradiated mushrooms exhibited a significant increase in serum 25(OH)D, closely mirroring the effect observed with supplementation, whereas no meaningful change was seen in the group consuming non-irradiated mushrooms. These results demonstrate that UV-induced vitamin D_2_ in mushrooms is bioavailable and effectively raises vitamin D status. The authors use this study to support the proposition that *A. bisporus*, as the most commonly consumed mushroom species, can serve as a practical and effective dietary source of vitamin D when subjected to UV exposure [25].

#### 4.1.16. *Aureobasidium pullulans*

*Aureobasidium pullulans*, a yeast-like fungus best known for producing the polysaccharide pullulan used in food and cosmetics, also yields β-(1,3)-(1,6)-glucan that promotes osteoblast differentiation through mechanisms involving Bone Morphogenetic Protein–7 and reduces bone loss in ovariectomized rat models of osteoporosis [65].

#### 4.1.17. *Wolfiporia extensa*

*Wolfiporia extensa* is valued in traditional Chinese medicine for its diuretic, sedative, and tonic properties, and is traditionally ascribed benefits for spleen and stomach function, as well as for resolving dampness. Pharmacological investigations have validated several of these traditional uses, demonstrating that *W. extensa* and its constituent polysaccharides possess potent antioxidant and immunomodulatory activities. Of particular relevance to bone tissue engineering, *W. extensa* has been shown to promote osteogenic differentiation while concurrently inhibiting osteoclastogenesis, effects that are mediated in part via modulation of key signaling pathways. This multifaceted biological activity positions *W. extensa* as a promising natural candidate for adjunctive therapies aimed at enhancing bone formation [72,73].

The mycelial extract of *W. extensa* inhibits osteoclast differentiation while concurrently promoting bone formation in cellular models, rendering it a promising candidate for further investigation as an anti-osteoporotic agent. In one study, osteoclast and osteoblast cultures were treated with an aqueous mycelial extract of *W. extensa* (WEMWE) to assess its effects on cellular differentiation, activity, and function. Functional assays demonstrated that WEMWE enhanced BMP-2-induced osteogenesis via activation of the Smad–Runx2 signaling axis. Concurrently, WEMWE suppressed RANKL-dependent osteoclast differentiation through inhibition of the c-Fos/NFATc1 pathway, an effect associated with blockade of ERK and JNK phosphorylation. These dual actions suggest that WEMWE may prevent and treat metabolic bone diseases, including osteoporosis, by contributing to the maintenance of bone homeostasis [72].

One article describes a study of hydroxyapatite (HAp) nanocomposites with *W. extensa* extract, stabilized with carboxymethylcellulose (CMC) and cerium oxide (CeO_2_), developed as an advanced antioxidant platform for bone regeneration. In vitro studies using osteoblastic and osteoclastic cell models demonstrated that the nanocomposites were biocompatible, enhanced osteogenic differentiation (increased ALP activity, increased expression of osteogenic transcription factors such as Runx2, and increased matrix mineralization), and simultaneously inhibited osteoclastogenesis and resorption activity. This material also reduced oxidative stress in cell cultures, consistent with the combined antioxidant effects of CeO_2_ and *W. extensa* components [73].

### 4.2. Summary of the Biological Activity of Selected Mushroom Species

Table 1 and Table 2 summarize mushroom-derived extracts, enriched fractions, and chemically characterized single compounds with documented relevance to osteoporosis prevention and therapy, evaluated in in vitro (Table 1) and in vivo (Table 2) models. Emphasis is placed on those fractions and isolated compounds exhibiting anti-osteoporotic activity, including stimulation of osteoblast differentiation and mineralization, inhibition of osteoclastogenesis and RANKL signaling, modulation of the OPG/RANKL axis and NF-κB/NFATc1 pathways, activation of osteogenic signaling (Wnt/β-catenin, BMP), antioxidant and anti-inflammatory effects, regulation of calcium homeostasis, and, in vivo, improvement of bone mineral density and trabecular structure. Collectively, the data compiled in these tables indicate that both complex mushroom-derived fractions and purified compounds target key molecular and cellular mechanisms underlying bone remodeling imbalance, supporting their potential as functional ingredients and lead structures for osteoporosis management.

## 5. Conclusions

In the scientific literature based on experimental studies and review papers, several complementary mechanisms have been described that may account for the observed beneficial effects of mushrooms on osteoporosis. These mechanisms involve modulation of calcium-phosphate homeostasis, regulation of osteoblast and osteoclast signaling pathways, reduction in oxidative stress, and modulation of the immune system, all of which collectively support the maintenance of normal bone mass. A mushroom-based diet may exert positive effects on bone health and aid in the management of osteoporosis. Mushrooms are a source of ergosterol, which, upon exposure to UV radiation, is converted into vitamin D_2_. This form of vitamin D can increase serum 25(OH)D concentrations and enhance intestinal calcium absorption, a process essential for bone mineralization. However, the current scientific literature still contains gaps regarding the impact of culinary processing on the stability and bioavailability of this vitamin, underscoring the need for further research. In addition to vitamin D_2_, mushrooms provide a variety of bioactive compounds, including protein necessary for maintaining muscle mass and calcium, a key structural component of bone. They are also a source of other trace elements important for bone metabolism, such as magnesium, selenium, and copper. Some experimental studies suggest that mushroom-derived mineral complexes may improve mineral bioavailability and exert beneficial effects in animal models of bone loss. Polysaccharides such as β-glucans may modulate the immune system and reduce inflammation, which is particularly relevant in the context of osteoporosis. β-glucans can influence immune cells by decreasing the production of pro-inflammatory cytokines (e.g., TNF α, IL 1β) and modifying RANK/RANKL/OPG signaling, a key regulatory system controlling osteoclast differentiation and activity. In vitro and animal studies have shown that extracts from certain mushroom species reduce bone resorption and promote osteogenesis. Antioxidants, such as phenolic acids present in mushrooms, counteract oxidative stress, which contributes to bone demineralization, and protect cells from damage. Additionally, some mushroom fractions influence anabolic osteoblast pathways, including Wnt/β-catenin and BMP signaling, potentially enhancing bone matrix synthesis and mineralization. Mushrooms also exert prebiotic effects, supporting a healthy gut microbiome that indirectly affects bone metabolism. Certain species contain phytoestrogen like compounds that may further benefit bone health, particularly in postmenopausal women.

Incorporating mushrooms into the diet may represent a reasonable, low cost, and safe component of osteoporosis prevention strategies, especially as a source of dietary fiber, antioxidants, and after appropriate UV exposure, vitamin D_2_. However, at the current stage of research, a mushroom rich diet should not be considered a substitute for established interventions such as antiresorptive or anabolic medications. Well-designed clinical trials using standardized mushroom preparations, defined dosing, safety assessments, and long term follow up are needed.

Although the promising properties of mushrooms require further clinical validation, they can already be regarded as a valuable dietary component for the prevention and maintenance of skeletal health.

## Figures and Tables

**Table 1:** Putative mechanisms underlying the anti-osteoporotic effects of mushrooms (in vitro study).

Mushroom Species	Research Material Used	Type of Study	Mechanism of Action	References
*Lentinula edodes*	Ethyl acetate extract	In vitro—osteoclastogenesis assay, RANKL-stimulated induction of NFATc1	Inhibition of osteoclast differentiation via attenuation of RANKL-stimulated NFATc1 induction	[2,30]
*Grifola frondosa*	Aqueous extract	In vitro—human osteoblastic osteosarcoma cell lines HOS58 and SaOS-2	Significantly increases alkaline phosphatase activity and promotes matrix mineralization bones	[41]
*Grifola gargal*	Aqueous extract	In vitro—assays (e.g., radical scavenging, inflammatory cytokine assays)	Antioxidant and anti-inflammatory activities that can indirectly protect bone by reducing oxidative stress and inflammation-driven bone resorption	[43]
*Ganoderma lucidum*	Ethanolic extract	In vitro—inhibition of osteoclast differentiation assessed in cell-based assays stimulated with RANKL and TNF-α	Inhibits osteoclastogenesis and bone resorption; reduces circulating osteocalcin in OVX models	[4,45,47]
	Ganomycin I	In vitro—RANKL osteoclast differentiation tests	Suppression of RANKL-induced osteoclast differentiation, inhibits phosphorylation of p38 MAPK, ERK, and JNK; suppresses induction of c-Fos and NFATc1; downregulates osteoclast markers (c-Src, TRAP, Cathepsin K, OSCAR, MMP-9, DC-STAMP), thereby blocking the osteoclastogenic program	[45,46]
	Ganoderic acid DM	In vitro—osteoclastogenesis assays in bone marrow cells and RAW 264D cell clones	Inhibits osteoclastogenesis by downregulating c-Fos and NFATc1, suppressing DC-STAMP expression and reducing osteoclast fusion	[22,46]
*Ganoderma sinense*	Aqueous extract	In vitro studies	Positive effect on the fracture healing process	[13]
*Ophiocordyceps sinensis*	Cs4–SeNP complex (polysaccharide-protein + selenium nanoparticles)	In vitro—MC3T3-E1 preosteoblastic cell line;	Endocytosed Cs4–SeNP stimulates Nox4-derived ROS—upregulates BMP-2 expression—activates canonical Smad signaling and Smad-independent p38 MAPK pathway—promotes osteoblast differentiation and mineralization.	[8,30,51]
*Cordyceps militaris*	Cordycepin	In vitro—enzymatic assays and studies using a cell model RAW 264.7	Inhibits RANKL-induced osteoclast differentiation; lowers expression of osteoclast marker genes and reduces TRAP activity.	[3,6]
*Pleurotus eryngii*	Aqueous extract	In vitro—human osteosarcoma cell line HOS (osteoblastic phenotype);	Stimulation of osteoblast differentiation and activity. Indirect suppression of osteoclastogenesis via elevated OPG (decoy receptor for RANKL) and direct reduction in TRAP(+) multinucleated cells and resorptive areas, leading to decreased bone resorption	[60]
*Pleurotus ostreatus*	Protein nano-extracts	In vitro—cell culture assays, human osteoblasts	Nano-formulations modulate inflammatory responses in macrophages while protecting and supporting osteoblast viability and function	[63]
*Pleurotus citrinopileatus*	Aqueous extract of isolated β-glucan	In vitro—osteoclastogenesis assays, osteoblast assays	Water-soluble β-glucans inhibit RANKL-stimulated osteoclast differentiation, reducing osteoclastogenic activity; Polysaccharide fractions promote osteoblast proliferation and increase ALP activity, shifting remodeling balance toward bone formation	[64]
*Pleurotus sajor-caju*	Isolated gluco-oligosaccharide (Ps–GOS)	In vitro—cell line: MC3T3-E1 preosteoblastic/osteogenic cell line	Wnt/β-catenin pathway activation and MAPK cascade stimulation. Increased osteoblast proliferation, accelerated maturation, and enhanced extracellular matrix mineralization; Ps-GOS acts as a pro-osteogenic polysaccharide that promotes osteoblast lineage commitment and function via canonical osteogenic signaling networks	[65]
*Auricularia auricula*	Peptide-calcium complex (AP-Ca)	In vitro studies	AP-Ca is absorbed by osteoblasts more readily than inorganic calcium, increasing intracellular Ca^2+^ and ALP activity, thereby promoting matrix synthesis and mineralization; AP-Ca lowers serum TRAP and downregulates pro-inflammatory genes associated with osteoclast activation, shifting remodeling toward formation	[12]
	Aqueous extract	In vitro—osteoclast differentiation assays	Aqueous extract suppresses RANKL-induced osteoclast differentiation by downregulating key transcriptional regulators (c-Fos, NFATc1) and osteoclast markers (TRAP, cathepsin K), and by blocking upstream ERK and JNK phosphorylation, thereby disrupting signaling required for osteoclast formation and function	[69]
*Aureobasidium pullulans*	β-(1,3)-(1,6)-glucan	In vitro—osteoblast precursor or osteoblastic cell assays	The β-glucan fraction promotes osteoblast differentiation, consistent with a direct pro-osteogenic effect on bone-forming cells	[65]
*Wolfiporia extensa*	Aqueous mycelial extract	In vitro—osteoblast and osteoclast cell models	Stimulates bone formation. The extract blocks RANKL-driven osteoclastogenesis by downregulating c-Fos and NFATc1 and reducing ERK/JNK phosphorylation, resulting in fewer active osteoclasts. Reduces oxidative stress	[72]

**Table 2:** Putative mechanisms underlying the anti-osteoporotic effects of mushrooms (in vivo study).

Mushroom Species	Research Material Used	Type of Study	Mechanism of Action	References
*Lentinula edodes*	Fruiting bodies exposed to UV	In vivo—ovariectomized and sham-operated rats	Increased mushroom-derived vitamin D_2_ leads to higher circulating 25(OH)D and enhanced calcium bioavailability	[37,38]
	Syringic acid	In vivo—ovariectomized mice model of postmenopausal osteoporosis	Reduced bone loss (higher BMD, better microarchitecture), reduced biochemical markers of bone resorption, improved bone formation indicators	[39]
*Grifola gargal*	Whole fruiting body powder—gargalols A-C	In vivo—measurement of a bone resorption biomarker	Reduces bone resorption by lowering deoxypyridinoline levels	[43]
*Ganoderma lucidum*	Ethanolic extract	In vivo—ovariectomized female Sprague-Dawley rats;	Inhibits osteoclastogenesis and bone resorption; reduces circulating osteocalcin in OVX models	[4,45,47]
*Ganoderma sinense*	Aqueous extract	In vivo studies	Positive effect on the fracture healing process	[13]
*Ophiocordyceps sinensis*	Cordymin	In vivo—alloxan-induced diabetic rodents	Suppression of ALP and TRAP activities; β-cell regeneration—lowered serum glucose and oxidative stress—secondary protection of bone formation and mineralization	[50]
	Cs4–SeNP complex (polysaccharide-protein + selenium nanoparticles)	In vivo—ovariectomized rodent model	Endocytosed Cs4–SeNP stimulates Nox4-derived ROS—upregulates BMP-2 expression—activates canonical Smad signaling and Smad-independent p38 MAPK pathway—promotes osteoblast differentiation and mineralization. Stimulates bone formation, inhibits osteoclast activity, restores bone microarchitecture in OVX model	[8,30,51]
*Cordyceps militaris*	Cordycepin	In vivo—animal model	Inhibits RANKL-induced osteoclast differentiation; lowers expression of osteoclast marker genes and reduces TRAP activity. Preserves bone tissue and mechanical resilience in vivo	[3,6]
	Ethanolic extract	In vivo—animal model	Limits inflammatory bone degradation by inhibiting osteoclast differentiation and activity	[56,57]
*Pleurotus eryngii*	Aqueous extract	In vivo—bilaterally ovariectomized rats	Stimulation of osteoblast differentiation and activity. Indirect suppression of osteoclastogenesis via elevated OPG (decoy receptor for RANKL) and direct reduction in TRAP(+) multinucleated cells and resorptive areas, leading to decreased bone resorption	[60]
*Pleurotus ferulae*	Pulsed UV-irradiated fruiting bodies	In vivo—ovariectomized mice	UV irradiation converts ergosterol/precursors in the fruiting bodies to vitamin D_2_, which is associated with greater suppression of osteoclast activity (lower PYD and NTX-I). Decreased serum PYD and NTX-I indicate attenuation of type I collagen breakdown and reduced bone resorption in animals. Metabolomics showed elevated serum levels of bone-relevant amino acids and metabolites after UV-PM consumption	[33]
*Pleurotus ostreatus*	UVB-irradiated powder	In vivo—mice	Dietary vitamin D_2_ from irradiated mushrooms increases systemic vitamin D status and calcium bioavailability, which promotes osteoblast/osteocyte numbers and function and reduces osteoclast abundance	[62]
*Antrodia camphorata*	Ethanolic extract	In vivo—ovariectomized SAMP8 mice	Triterpenoids in ACAE likely suppress osteoclastogenesis by reducing RANKL expression, thereby inhibiting pro-osteoclast signaling; Polysaccharides promote osteoblast differentiation and osteogenesis, supporting matrix formation. Ergosterol may act as a vitamin D precursor, enhancing osteoblastic differentiation and calcium homeostasis	[17]
*Agaricus bisporus*	Powdered whole-fruiting-body	In vivo—*Coturnix coturnix japonica*	Improvement of the biomechanical properties of bones; Increasing bone shear strength	[70]
	Glucosamine hydrochloride	In vivo— *Brachydanio rerio* larvae	GAH promoted osteogenesis in larvae and enhanced fin regeneration in adults; upregulated bone marker genes and acted via bone morphogenetic protein signaling, supporting roles in cartilage/proteoglycan synthesis and skeletal repair	[71]
*Aureobasidium pullulans*	β-(1,3)-(1,6)-glucan	In vivo—ovariectomized rat	The β-glucan fraction promotes osteoblast differentiation, consistent with a direct pro-osteogenic effect on bone-forming cells	[65]

## Data Availability

No new data were created or analyzed in this study. Data sharing is not applicable to this article.
